# Sensorineural Hearing Loss as the Prominent Symptom in Meningeal Carcinomatosis

**DOI:** 10.3390/curroncol28050281

**Published:** 2021-08-25

**Authors:** Xiaoqin Huang, Yu Jia, Lidong Jiao

**Affiliations:** Department of Neurology, Xuanwu Hospital, Capital Medical University, Beijing 100053, China; huangxqxw@126.com (X.H.); jiayu_2219@163.com (Y.J.)

**Keywords:** meningeal carcinomatosis, sensorineural hearing loss, cranial nerves, primary tumor, therapy

## Abstract

Sensorineural hearing loss (SNHL) has been reported rarely in patients with meningeal carcinomatosis (MC). We summarized the clinical data of eight MC patients with SNHL and 35 patients reported from publications. In the eight patients with SNHL, the medium onset age was 48 (range from 37 to 66) years and six (75%) were male. Seven (87.5%) suffered from headaches as the initial symptom, and they experienced SNHL during the first two months after the occurrence of headaches (0.5 to 2 months, average 1.5 months). The audiogram configuration was flat in three patients (37.5%) and showed total deafness in five patients (62.5%). The damage of cranial nerves VI (abducens) was observed in six patients (75%), and four patients (50%) had cranial nerves VII (facial) injury during the disease course. The percentage of damage of cranial nerves was higher than the patients without SNHL (VIth, 75.0% vs. 13.3%, *p* = 0.002 and VIIth 50.0% vs. 6.7%, *p* = 0.012). Four (50%) patients suffered from lung adenocarcinoma as primary tumor, two (25%) experienced stomach adenocarcinoma, one had colon cancer, and one patient was unknown. The symptom of SNHL improved after individualized therapy in four patients (focal radiotherapy and chemotherapy for three patients and whole brain radiotherapy for one patient), but all passed away from 2 to 11 months after diagnosis. Total deafness and flat hearing loss in audiogram were the common types of SNHL resulting from MC. MC patients with SNHL were more likely to suffer from the damage of other cranial nerves, especially to cranial nerves VI and VII. Treatment might improve SNHL, but not improve the case fatality rate.

## 1. Introduction

Meningeal carcinomatosis (MC), also known as carcinomatous meningitis or leptomeningeal carcinomatosis (LMC), is characterized by diffuse infiltration of cancer cells to the leptomeninges and cerebrospinal fluid (CSF) from solid tumors, commonly from lung and breast cancer, melanoma, and lymphoma [1,2]. The symptoms of MC are typically widespread, involving central and peripheral nervous systems. 

The damage of cranial nerves III (oculomotor), IV (trochlear), VI (abducens), and VII (facial) occurred frequently during the disease course [3]. However, cranial nerves VIII (vestibulocochlear) lesions had been reported rarely in patients with MC [3]. In our study, we analyzed the clinical data of eight MC patients in our hospital, and 35 MC patients identified through a review of the literature with sensorineural hearing loss (SNHL) as the prominent symptom, and compared the clinical characteristics of patient with SNHL and without SNHL.

## 2. Materials and Methods

### 2.1. Patients

The clinical information of 38 MC patients in Department of Neurology, Xuanwu Hospital, Capital Medical University from September 2013 to September 2019, were collected, retrospectively. All patients underwent the lumbar puncture, and malignant cells were observed in CSF analysis to define the diagnosis of MC. We performed a long-term follow-up investigation for these 38 patients. Among these patients, 10 patients had the complaint of hearing loss. While 2 patients had a history of deafness for many years, the remaining 8 patients developed hearing loss during the disease course. All 8 patients were diagnosed as sensorineural hearing loss (SNHL) by pure tone audiometry (PTA) and were included in this study. Written informed consent was obtained from all patients’ dependents for collection of clinical information and the publication of articles.

### 2.2. Identification of SNHL

Pure tone audiometry (with air and bone conduction thresholds) differentiated conductive from sensorineural loss and defined the severity of hearing loss, and all 8 patients met the diagnostic criteria of sensorineural hearing loss (SNHL). Audiometry results were analyzed using four contiguous frequency pure-tone average (500 Hz, 1000 Hz, 2000 Hz, and 4000 Hz). All hearing loss was defined as any hearing loss above 20 dB, ranging from mild to complete in severity (Mild hearing loss, 20–34 dB; Moderate hearing loss, 35–49 dB; Moderately severe hearing loss, 50–64 dB; Severe hearing loss, 65–79 dB; Profound hearing loss, 80–94 dB; Complete hearing loss, ≥95 dB) [4].

### 2.3. Clinical Data

We collected and analyzed the detailed clinical data, including demographics, clinical manifestation, auxiliary examination, treatments, and prognosis. All patients were followed 6 months to 3 years after discharge. Treatment effect was evaluated by the Modified Rankin score (mRS), progression-free survival (PFS), and overall survival (OS). The outcome of SNHL was evaluated by pure tone audiometry (PTA) with average thresholds ≥ 15 dB during follow-ups defined as efficacious. Then, we compared the clinical characteristics, such as the ratio of male to female, age, initial symptom, other symptoms, and damage of cranial nerves, of patients with and without SNHL.

### 2.4. Systematic Review and Data Extraction

We performed a PubMed search to identify the relative publications regarding meningeal carcinomatosis with sensorineural hearing loss by using the keywords of meningeal carcinomatosis (carcinomatous meningitis or leptomeningeal carcinomatosis) and sensorineural hearing loss. A total of 35 patients from 27 published articles were included in this study. We summarized the clinical data, including age, gender, and side of SNHL, as well as the primary tumor of MC.

### 2.5. Statistical Analysis

All statistical analyses were performed with the SPSS version 19.0 (SPSS Inc, Chicago, IL, USA). Univariate analysis of nominal and interval variables were performed using the Mann–Whitney test, McNemar test, and Pearson’s Chi-squared or Fisher exact test, respectively.

## 3. Results

### 3.1. Demographics

Among the 38 MC patients from our hospital, 20 (52.6%) were male, and the median onset age of MC was 45 (range from 15 to 66) years. Nine patients had a history of hypertension, five had diabetes, two patients had coronary heart disease, and one patient suffered from chronic valvulopathy. Of 38 MC patients, eight patients (21.1%) developed sensorineural hearing loss (SNHL). In these eight patients, the medium onset age of MC was 48 (range from 37 to 66) years and six of them (75%) were male. Compared the demographics information of patients with and without SNHL, there was no statistical difference of the gender and age between two groups (Table 1).

### 3.2. Clinical Characteristics

In the 38 MC patients, initial symptoms comprised headache (27 patients (71.1%)), visual impairment (6 patients (15.8%)), limb weakness and numbness (2 patients (5.3%)) and, less commonly, epilepsy onset (2 patients (4.8%)) (Table 1).

Eight patients developed SNHL in early stage of disease (six with bilateral SNHL and two with right sided SNHL), seven (87.5%) suffered from headaches as the initial symptom. The patients experienced SNHL during the first two months after occurrence of the headaches (0.5 to 2 months, average 1.5 months). The damage of cranial nerves III (oculomotor) occurred in two patients (25%), cranial nerves VI (abducens) in six patients (75%), and cranial nerves VII (facial) in four patients (50%), and the percentage of damage of cranial nerves was higher than the patients without SNHL (VIth, 75.0% vs. 13.3%, *p* = 0.002 and VIIth 50.0% vs. 6.7%, *p* = 0.012).

### 3.3. Auxiliary Examination

Subjective pure tone audiometry (PTA) and the air conduction thresholds of eight patients were shown in Table 2. The audiogram configuration was flat in three patients (37.5%) and showed total deafness in five patients (62.5%).

All patients underwent the lumbar puncture. CSF pressure was 270 (ranging from 50 to 330) mmH_2_O, and 33 (86.8%) patients showed increased pressure. Routine CSF tests showed elevated white blood cell counted in 34 (89.5%) patients (16 (range from 5 to 126) ∗ 106/L), and increased protein in 27 (71.1%) patients (66 (range from 24 to 443) mg/dL, normal ranges 15–45 mg/dL). A total of 16 (42.1%) patients indicated a decreased glucose level in CSF (33.5 (range from 12.1 to 86) mg/dL, normal ranges 45–80 mg/dL). Additionally, CSF IgG showed increased level in 28 patients (73.7%) (7.8 (range from 2.7 to 125) mg/dL, normal ranges 0.48–5.86 mg/dL).

For the eight patients with SNHL, increased CSF pressure was observed in seven patients (87.5%), and six patients had high status of more than 330 mmH_2_O. Seven patients (87.5%) had elevated white blood cell counts (15.5 ((range from 5 to 61) cells/mcL) and increased protein concentration (94.5 (range from 34 to 250) mg/dL). CSF chlorides and oligoclonal Bands (OB) tests exhibited normal results. Cytology analysis identified malignant cells in all patients.

Brain gadolinium-enhanced MRI showed different degrees of meningeal reinforcement in 26 (68.4%) patients, and for the patients with SNHL, brain MRI revealed that the thickening and enhancement of both vestibulocochlear nerves in four (50%) patients (cases 2, 4, 6, and 7). All these four patients with VIIIth nerve involvement developed bilateral SNHL, and the magnitude of hearing loss was worse than those patients without VIIIth nerve enhancement on brain MRI (Table 2). Among these four patients, the patterns of hearing loss was total deafness in three patients and another was flat.

According to past history of tumors and screening, the primary tumors of MC included lung adenocarcinoma (16 patients, 42.1%), lung small cell cancer (5 patients, 13.2%), stomach adenocarcinoma (4 patients, 10.5%), breast adenocarcinoma (3 patients, 7.9%), non-Hodgkin’s lymphoma (2 patients, 5.3%), Melanomas (1 patient, 2.6%), Teratocarcinoma (1 patient, 2.6%), colon cancer (1 patient, 2.6%) and unknown/adenocarcinoma (5 patients, 13.2%). For patients with SNHL, four (50%) patients suffered from lung adenocarcinoma, two (25%) experienced stomach adenocarcinoma, one (12.5%) had colon cancer and one (12.5%) patient did not identify the primary tumor (Table 2).

### 3.4. Treatment and Prognoses

Most patients were treated with chemotherapy or radiotherapy according to the different condition. The patients were followed up 1 month to 1 year through consultation or telephone calls after discharged; five patients were lost to follow-up and the remaining (33 patients) had accomplished. Despite chemotherapy and/or radiotherapy, 28 (73.7%) patients’ condition quickly deteriorated, and they passed away between 1 and 6 months after diagnosis; the remaining died between 6 months and 1 year after diagnosis.

The median PFS was 3.35 months (95% CI 2.91–4.73 months), and the median OS of all patients was 5.25 months (95% CI 4.80–6.63 months). Considering deaths from all causes, 29 patients died from dyscrasia or neurologic involvement caused by MC, and others died of acute coronary syndrome (two patients), severe pneumonia (one patient) or acute cerebral hemorrhage (one patient).

For the patients with SNHL, symptoms of hearing loss were improved after therapy in 4 (50%) patients (focal radiotherapy and chemotherapy for three patients (case 1, 5 and 8) and whole brain radiotherapy for one patient (case 3)). The median PFS was 2.25 months (95% CI 1.19–5.31 months), and the median OS of MC patients with SNHL was 4.30 months (95% CI 3.52–7.08 months).

### 3.5. Systematic Literature Review

As seen in Table 3, the clinical data of 35 MC patients with SNHL reported in the literature were collected [5,6,7,8,9,10,11,12,13,14,15,16,17,18,19,20,21,22,23,24,25,26,27,28,29,30,31]. The age was 61 (42, 77) years and ratio of male to female was 4:3. In total, 71.4% of patients developed bilateral SNHL. Hearing loss as the initial symptom was found in eight patients (22.9%) and it often accompanied by symptoms of vertigo (24 patients, 68.6%), tinnitus (10 patients, 28.6%) and unsteadiness (13 patients, 37.1%). Damage of cranial nerves VIIth was not common in MC patients with SNHL, and seven patients (20%) suffered from facial palsy during the disease course. Moreover, brain MRI revealed swelling and increased signal intensity on T2WI/FLAIR images of unilateral or bilateral vestibulocochlear nerves in 10 patients (28.6%) and involvement of internal auditory meatus (IAM) in 17 patients (48.6%).

In terms of the primary tumor leading to MC, lung cancer (22.9%, 8/35), esophagus adenocarcinoma (14.2%, 5/35), and gastric cancer (14.2%, 5/35) were the major causes related to the development of MC. In addition, we collected the treatments and outcomes of MC patients with SNHL from publications. Treatment options were very limited. A majority of patients continued to deteriorate, and only two cases responded well to the treatments of a combination of radiation therapy and chemotherapy [25,31].

## 4. Discussion

MC, defined as the invasion of subarachnoid spaces by malignant cells from a primitive cancer, is a rare and complex neurological disorder. It tends to be more susceptible to elderly and middle-aged patients, which extensively affects nervous systems [3]. SNHL is an atypical and uncommon symptom in patients with MC. In the present study, we collected the clinical information of eight MC patients suffering from SNHL during the disease course. In total, 87.5% patients presented with headaches as the initial symptom. Hearing loss was often the subsequent symptom after headaches, with an interval of 1.5 months. Moreover, MC patients with SNHL were more likely to develop the damage of other cranial nerves, especially to the cranial nerves VIth and VIIth, but fewer problems on urinary retention, as well as limb weakness and numbness.

We summarized the clinical information of a total of 35 MC patients with SNHL reported in the literature [5,6,7,8,9,10,11,12,13,14,15,16,17,18,19,20,21,22,23,24,25,26,27,28,29,30,31]. A majority of patients suffered from bilateral SNHL; fewer developed one-sided hearing loss. As for the primary tumor leading to MC, lung cancer was the main cause, followed by esophagus adenocarcinoma, and gastric cancer. Due to limited access to audiogram data of some of the literature, the type of SNHL from most publications was total deafness. In our study, the audiogram configuration was flat in three patients (37.5%) and showed total deafness in five patients (62.5%), which was consistent with the literature reports.

For the treatment of MC, a combination of radiotherapy and intrathecal chemotherapy were the first-line therapy. However, disease severity and rapid deterioration of MC contributed to a poor prognosis. The median survival of untreated patients was about 4–6 weeks, and in some patients can be prolonged to 4–6 months for survival after aggressive treatment [3,21]. In our study, though half of patients had improved symptoms of SNHL after treatment, the median OS was only 5.05 months. However, most encouragingly, two cases reported by de Mones del Pujol E and Nakashima K et al. responded very well to the treatments of radiation therapy associated with chemotherapy (Methotrexate or Pembrolizumab) [25,31]. Thus, further studies need to focus on the rare MC cases with good prognosis, and explore the best individualized treatment to prolong the survival time of patients.

## 5. Conclusions

SNHL is an atypical symptom in MC. Total deafness and flat hearing loss in audiogram were the common types of SNHL caused by MC. MC patients with SNHL were more likely to suffer from the damage of cranial nerves, especially to cranial nerves VIth and VIIth. Treatments might improve SNHL, but not improve the case-fatality rate. Thus, we should be alert to the possibility of MC in patients with SNHL, and the suspected patients will benefit from early brain MRI, cytology in CSF, as well as the tumor screening.

## Figures and Tables

**Table 1:** The comparison of clinical characteristics of patients with SNHL and without SNHL.

Variable	Patients with SNHL (*n* = 8)	Patients without SNHL (*n* = 30)	*p* Value *
Male (%)	6 (75.0%)	14 (46.7%)	0.238
Age (median (range)] (years)	48 (37, 66)	45 (15, 64)	0.276
Initial symptoms			
headache	7 (87.5%)	20 (66.7%)	0.395
visual impairment	1 (12.5%)	5 (16.7%)	1.000
Other symptoms			
dizziness	2 (25.0%)	9 (30.0%)	1.000
vomiting	5 (62.5%)	20 (66.7%)	1.000
diplopia	3 (37.5%)	16 (53.3%)	0.693
epileptic convulsions	2 (25.0%)	11 (36.7%)	0.689
mental disorders	2 (25.0%)	7 (23.3%)	1.000
impaired consciousness	3 (37.5%)	9 (30.0%)	1.000
urinary retention	0	5 (16.7%)	0.337
limb weakness and numbness	0	7 (23.3%)	0.307
Damage of cranial nerves			
III (oculomotor)	2 (25.0%)	2 (6.7%)	0.189
VI (abducens)	6 (75.0%)	4 (13.3%)	0.002
VII (facial)	4 (50.0%)	2 (6.7%)	0.012
Primary tumors			
lung adenocarcinoma	4 (50.0%)	12 (40.0%)	0.698
stomach adenocarcinoma	2 (25.0%)	2 (6.7%)	0.189
breast adenocarcinoma	0	3 (1.0%)	0.587
colon cancer	1 (12.5%)	0	0.211

* Results of Mann–Whitney Test or Fisher Exact Test.

**Table 2:** The characteristic of pure tone audiometry in eight patients with SNHL.

Case	Air Conduction Threshold (dB)															
	Right Ear								Left Ear							
	250 Hz	500 Hz	1000 Hz	2000 Hz	4000 Hz	8000 Hz	PTA (Hz)	Magnitude	250 Hz	500 Hz	1000 Hz	2000 Hz	4000 Hz	8000 Hz	PTA (Hz)	Magnitude
1	75	65	70	75	90	105	75	S	85	80	85	75	90	85	83	P
2	105	105	100	>120	>120	>120	111	C	75	80	85	80	90	105	84	P
3	90	85	85	80	75	75	81	P	10	15	10	15	15	20	14	N
4	110	>120	>120	>120	>120	>120	120	C	65	70	55	60	65	50	63	MS
5	55	60	55	55	50	45	55	MS	30	35	45	55	60	70	49	M
6	75	100	90	>120	100	105	103	C	>120	>120	>120	>120	>120	>120	120	C
7	105	110	>120	100	>120	100	113	C	70	75	80	80	85	90	80	P
8	55	50	60	55	70	65	59	MS	10	5	5	10	5	10	6	N

PTA: Pure-tone average; N: Normal; M: Moderate hearing loss; MS: Moderately severe hearing loss; S: Severe hearing loss; P: Profound hearing loss; and C: Complete hearing loss.

**Table 3:** The summary of the clinical data of 35 reported MC patients from publications and the present 8 cases with SNHL.

Author/Year		Age (Years)	Gender	Side of SNHL	Primary Tumor	Treatment	Outcome
Shen TY et al. [5]/2000		51	M	B	Lung cancer	NA	Died 1 year after diagnosis
Uppal HS et al. [6]/2001		61	F	B	Breast cancer	Dexamethasone and tamoxifen	Died 11 weeks after presentation.
Currie L et al. [7]/2001		49	F	B	Melanoma	Dexamethasone	Died 3 weeks after her original presentation
Boukriche Y et al. [8]/2002		59	M	B	Bladder transitional cell carcinoma	Chemotherapy	NA
Wagemakers M et al. [9]/2005		52	M	B	Esophagus adenocarcinoma	Radiotherapy of the skull base	Died 16 weeks after the onset of deafness.
Testoni S et al. [10]/2005		53	M	B	Melanoma	NA	NA
Jeffs GJ et al. [11]/2006		66	F	B	Melanoma	Steroid, urgent adjuvant radiotherapy	Her hearing loss did not improve. Died 2 weeks after the onset of hearing loss.
Jariengprasert C et al. [12]/2006		64	F	B	Unknown	She refused any kind of treatment and consented to go back home.	Died 10 weeks after the onset of symptoms.
Suzuki T et al. [13]/2006		60	F	B	Rectum adenocarcinoma	NA	Died 4 months after the diagnosis of MC.
Lai TH et al. [14]/2006		66	M	B	Lung cancer	The family refused further treatment.	The patient was discharged the next day.
Baba S et al. [15]/2006		59	M	R	Gastric cancer	NA	The patient became unconscious, and died 3 month after onset
Koda H et al. [16]/2008		63	M	B	Esophagus adenocarcinoma	NA	Died of pneumonia 5 months after the onset of hearing loss.
Vitaliani R et al. [17]/2009		59	F	R	Over carcinoma	Supportive therapy	She eventually became comatose and died 20 days after admission.
Gu CS et al. [18]/2009		59	M	L	Lung adenocarcinoma	Chemoradiotherapy	NA
Mourgela S et al. [19]/2009		56	M	B	Lung small cell cancer	Whole brain radiation therapy and methotrexate intrathecally	NA
Marchese MR et al. [20]/2010		56	M	R	Pancreas cancer	NA	Died few days after the diagnosis of MC
		64	F	B	Unknown	Chemotherapy and stereotassic radiotherapy	Until now without clinical improvement.
Ohno T et al. [21]/2010		62	M	B	Gastric cancer	Radiation therapy to the whole brain and spine, chemotherapy with S-1 and paclitaxel	Deafness did not improve. Died 12 weeks after the onset of deafness.
Bruce BB et al. [22]/2010		65	M	B	Colorectal cancer	Whole brain radiation	The patient’s clinical status deteriorated rapidly to death.
Kato Y et al. [23]/2012		77	F	B	Colon cancer	NA	Died from cachexia 6 weeks after admission to our hospital.
Öztürk M et al. [24]/2014		44	M	B	Duodenum adenocarcinoma	NA	NA
de Mones del Pujol E et al. [25]/2014		76	F	R	Breast Adenocarcinoma	Radiotherapy to the whole brain	NA
		60	F	L	Breast and lung cancers	Radiotherapy to the whole brain	Died 1 month later with a bilateral encasement of the lower cranial nerves resulting from MC.
		42	F	R	Unknown	Fractionated radiation therapy to the whole brain [46 Gy] was performed as an emergency, associated with intrathecal chemotherapy [methotrexate]	Eight weeks after treatment, hearing loss was improved. Died disease-free 6 years later after falling downstairs owing to her residual unsteadiness.
		61	F	R	Lung cancer	whole-brain radiotherapy	Died of multiple metastases 14 months after hearing loss.
		67	M	R	Melanoma	Chemotherapy with Temodal and whole-brain radiotherapy	NA
		77	M	R	Unknown	No	Died 2 days after diagnosis, 2 months after the onset of symptom.
Rakusic Z et al. [26]/2015		60	F	B	Gastric cancer	No	Died 6 weeks from the first symptoms.
Adams M et al. [27]/2015		52	M	B	Esophagus adenocarcinoma	A palliative approach	Died at home 21 days after presenting for assessment.
		71	M	B	Esophagus adenocarcinoma	A palliative approach	Died 42 days after initial presentation.
		43	M	B	Esophagus adenocarcinoma	NA	The patient continued to deteriorate and died 1 month following initial presentation.
Costentin G et al. [28]/2018		63	F	B	Lung small cell cancer	Intravenous administration of high-dose corticosteroids and palliative care	Died a few days later.
Kimura A et al. [29]/2019		66	M	B	Gastric cancer	NA	Died 46 days after the first onset of hearing loss.
Tanaka T et al. [30]/2020		68	M	B	Gastric cancer	No	Died approximately two months after the symptom onset.
Nakashima K et al. [31]/2020		66	F	B	Lung adenocarcinoma	Whole brain radiation therapy (30 Gy: 3 Gy × 10), chemotherapy with Pembrolizumab	Consciousness recovered. Brain MRI and CSF results were improved, although deafness remained. A total of 23 months have passed since the diagnosis of MC, and chemotherapy is ongoing (30 cycles) without disease progression.
The present cases	Case 1	38	F	B	Lung adenocarcinoma	Focal radiotherapy and chemotherapy	Hearing loss improved, symptoms worse 5 weeks after treatment and died approximately four months after the symptom onset.
	Case 2	51	M	B	Lung adenocarcinoma	Radiotherapy and palliative care	Died 50 days after the first onset of hearing loss.
	Case 3	66	M	R	Stomach adenocarcinoma	Whole brain radiotherapy	Hearing loss improved, cancer recurred 2 month after treatment and died 6 months after hearing loss.
	Case 4	37	M	B	Lung adenocarcinoma	Chemotherapy	Hearing loss did not improve. Died 15 weeks after the onset.
	Case 5	56	M	B	Unknown	Radiotherapy and chemotherapy	Hearing loss improved, disease-free 8 months later after acute coronary syndrome.
	Case 6	54	F	B	Lung adenocarcinoma	Radiotherapy to the whole brain	The patient’s clinical status deteriorated rapidly to death.
	Case 7	38	M	B	Stomach adenocarcinoma	Radiotherapy and chemotherapy	Died 17 weeks after onset.
	Case 8	45	M	R	Colon cancer	Focal radiotherapy and chemotherapy	Hearing loss recovered and brain MRI result was improved. Cancer recurred 6 month after treatment and died 8 month after initial presentation.

M: Male; F: Female; B: bilateral; R: Right; L: Left; and NA: Not available.

## Data Availability

Data is contained within the article.
